# Percutaneous closure of ventricular septal rupture after myocardial infarction: a case report of competing timelines in multifactorial shock

**DOI:** 10.1093/ehjcr/ytag253

**Published:** 2026-04-20

**Authors:** Thomas A Cook, Simone K Schumaecker, Anjani Patibandla, Nicholas J Valle, Matthew R Summers

**Affiliations:** Doctor of Medicine Program, Macon & Joan Brock Virginia Health Sciences at Old Dominion University, Eastern Virginia Medical School, Norfolk, VA 23507, USA; Doctor of Medicine Program, Macon & Joan Brock Virginia Health Sciences at Old Dominion University, Eastern Virginia Medical School, Norfolk, VA 23507, USA; Doctor of Medicine Program, Macon & Joan Brock Virginia Health Sciences at Old Dominion University, Eastern Virginia Medical School, Norfolk, VA 23507, USA; Department of Internal Medicine, Macon & Joan Brock Virginia Health Sciences at Old Dominion University, Norfolk, VA 23507, USA; Department of Structural Cardiology, Sentara Medical Group, Norfolk, VA 23507, USA

**Keywords:** Myocardial infarction, Ventricular septal rupture, Intra-aortic balloon pump, Cardiogenic shock, Case report

## Abstract

**Background:**

Ventricular septal rupture (VSR) following acute myocardial infarction is a rare but deadly complication with mortality rates exceeding 90% when left untreated. Coronary ischaemia leads to myocardial necrosis and friability resulting in septal rupture, most commonly 3–5 days post-infarction. The sudden development of a left-to-right shunt causes volume overload in the right ventricle, pulmonary vasculature, and left atrium, precipitating biventricular failure, and cardiogenic shock. Management requires careful consideration of timing and modality of closure along with stabilization with mechanical circulatory support in severe cases.

**Case summary:**

A 65-year-old female presented with anterior ST-segment elevation myocardial infarction complicated by VSR diagnosed the same day, cardiogenic shock, and progressive multiorgan dysfunction. Following intra-aortic balloon pump (IABP) placement and percutaneous coronary intervention to the left anterior descending artery, she experienced ventilator-associated pneumonia, ventricular fibrillation arrest, and acute renal failure requiring continuous renal replacement therapy. Despite inotropic support and IABP, the patient remained in cardiogenic shock. Given her persistent haemodynamic instability, the decision was made to proceed with delayed percutaneous VSR repair. On hospital day 11, she underwent percutaneous VSR closure with a 24-mm Amplatzer occluder device, resulting in immediate haemodynamic improvement leading to successful IABP removal. In spite of VSR repair, the patient's pulmonary sepsis worsened precipitating left ventricular dysfunction requiring Impella CP placement. Despite mechanical circulatory support, multiorgan failure ensued and the patient ultimately expired.

**Discussion:**

This case highlights the challenges of managing post-infarct VSR with delayed percutaneous device closure in the setting of mixed cardiogenic and septic shock.

Learning points

- Delayed percutaneous repair of post-infarct VSR may be feasible in selected patients, allowing time for infarcted tissue to mature and better anchor closure devices.
- Delaying VSR closure can be challenging in patients with multifactorial shock, as competing physiologic demands may limit myocardial recovery.

## Introduction

Optimal timing of post-infarct ventricular septal rupture (VSR) repair remains controversial. Immediate repair within 7 days of infarction, usually 24–72 h after VSR diagnosis, is required in patients with refractory cardiogenic shock. However, feasibly delaying intervention to ≥7 days after infarction allows infarcted myocardium to fibrose, strengthening tissue for suturing, or device anchoring and improving procedural success with reported mortality reduction to 32–44%.^1–3^ Unfortunately, many patients cannot be stabilized long enough to reach the delayed repair window, even with mechanical circulatory support (MCS). Current American Heart Association and European Society of Cardiology guidelines recommend urgent VSR closure in all patients, with surgical repair being standard and percutaneous closure as a viable alternative, while recognizing that select patients may benefit from delayed repair to improve procedural outcomes.^4,5^ This case illustrates the clinical and logistical considerations for pursuing delayed percutaneous VSR closure. Specifically, the need for multidisciplinary decision-making, the management of multifactorial shock contributing to haemodynamic instability, and the challenges associated with delaying intervention in a hypoxic and intubated patient.

## Summary figure

Timeline of the patient’s clinical course across cardiac, mechanical circulatory support, respiratory, renal, and infectious domains. Abbreviations: HD, hospital day; POD, postoperative day; STEMI, ST-elevation myocardial infarction; VSR, ventricular septal rupture; VF, ventricular fibrillation; IABP, intra-aortic balloon pump; MCS, mechanical circulatory support; SBT, spontaneous breathing trial; AKI, acute kidney injury; CRRT, continuous renal replacement therapy.

**Figure:**
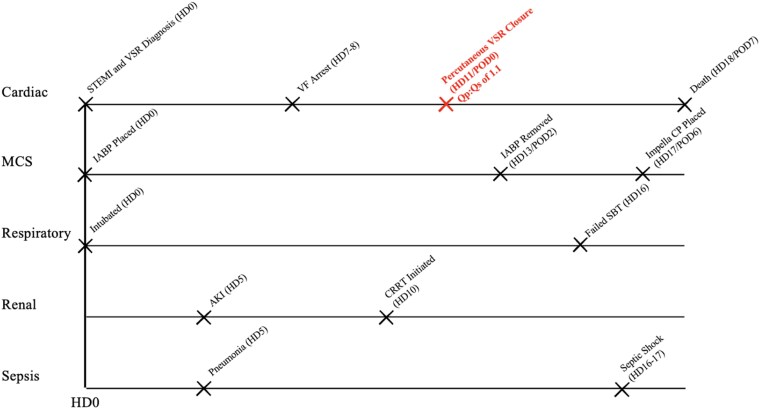


## Case presentation

**Figure 1:**
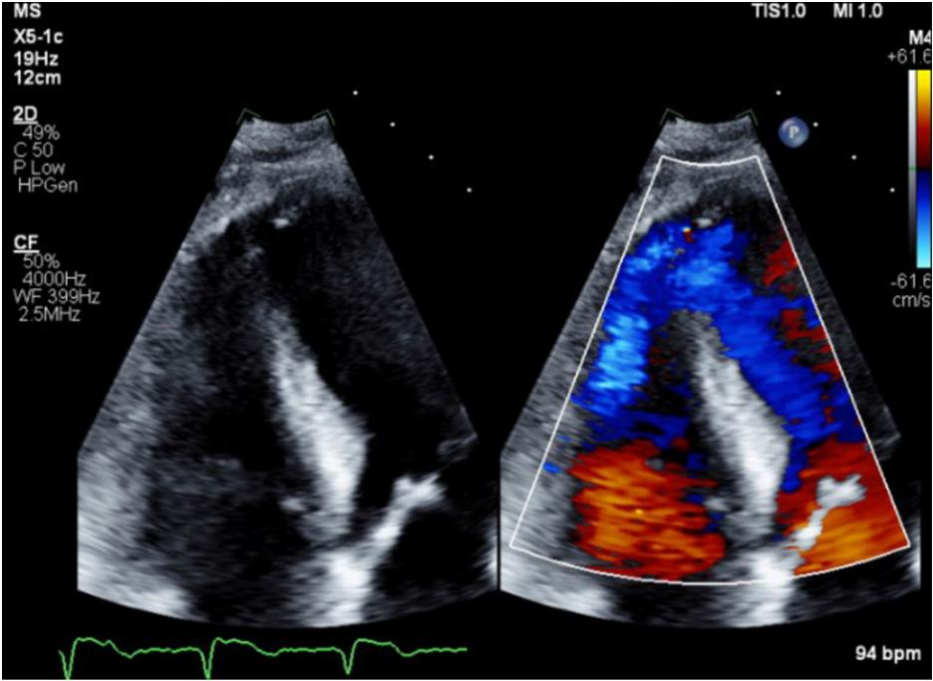
Transthoracic echocardiography demonstrating apical ventricular septal rupture (VSR). Apical four-chamber view with colour Doppler imaging shows a defect in the apical interventricular septum with continuous left-to-right shunting.

A 65-year-old female with a history of hypertension, uncontrolled type 2 diabetes, and obesity presented to a local emergency department with acute onset of shortness of breath and chest discomfort. She appeared acutely ill and diaphoretic, with a blood pressure of 202/152 mmHg, heart rate of 160 bpm, respiratory rate of 40 breaths per minute, and oxygen saturation of 92% on room air. Physical examination was notable for bilateral rales and laboratory tests revealed elevated hs-T-troponin of 3118 ng/L. An electrocardiogram showed anterolateral ST-segment elevations and chest x-ray displayed an enlarged cardiac silhouette with increased pulmonary vascular markings. The patient was intubated for acute hypoxic respiratory failure and transferred to a tertiary care centre. Coronary angiography demonstrated subtotal occlusion of the ostial-to-proximal left anterior descending artery with TIMI 1 flow, and she underwent emergent percutaneous coronary intervention (PCI) with aspiration thrombectomy and deployment of a drug-eluting stent. Left ventriculography revealed a reduced left ventricular ejection fraction (LVEF) of 40%, an apical muscular VSR, and oxygen saturation step-up from the right atrium (60%) to the right ventricle (85%) consistent with left-to-right shunting. The VSR was diagnosed the same day as the myocardial infarction by left ventriculography and subsequently visualized on transthoracic echocardiography (*Figure 1*). Despite successful revascularization, she developed worsening cardiogenic shock, and an intra-aortic balloon pump (IABP) was placed with initiation of norepinephrine for haemodynamic support.

**Figure 2:**
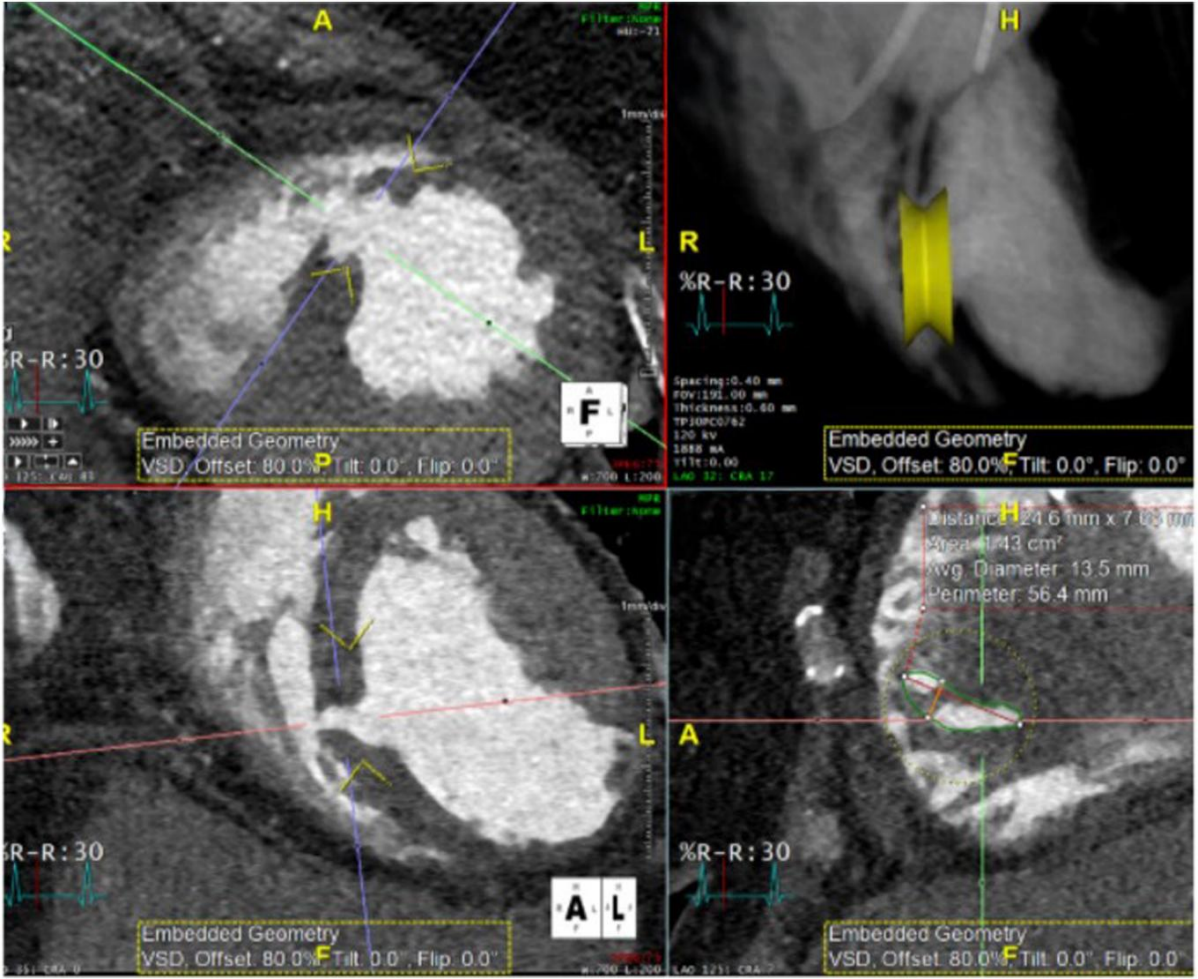
Preprocedure cardiac computed tomography displaying a 4 mm apical VSR and 3D rendering of planned VSR closure device.

Following PCI and IABP placement, the patient remained intubated in the cardiovascular intensive care unit (CICU) and structural cardiology and cardiothoracic surgery were consulted for percutaneous vs. surgical VSR closure. Overall, she was deemed a poor candidate for immediate surgical repair. The CICU team continued medical management with inotropes and structural cardiology proposed delayed closure to promote strengthening of peri-infarct tissue. Cardiac computed tomography (CT) with 3D reconstruction was obtained for procedural planning (*Figure 2*). The patient subsequently developed ventilator-associated pneumonia with *Burkholderia cepacia*, multiple episodes of ventricular fibrillation arrest on hospital days 7 and 8, and renal failure requiring continuous renal replacement therapy initiation on hospital day 10. Her co-oximetry at that time was 43.7%, reflective of severe low flow state consistent with cardiogenic shock.

**Figure 3:**
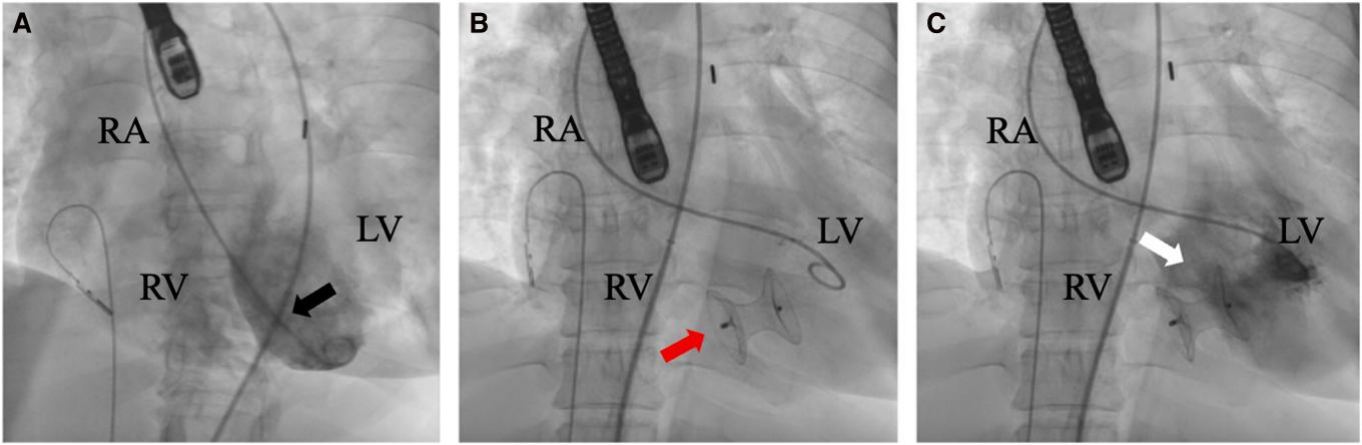
Fluoroscopic images demonstrating stages of percutaneous VSR closure. *(A)* Left anterior oblique projection demonstrating contrast passage through the apical interventricular septum consistent with left-to-right shunting across the VSR (black arrow). *(B)* Deployment of a 24-mm Amplatzer occluder device across the interventricular septum (red arrow), demonstrating positioning of the device across the septal defect. *(C)* Left ventriculography following device deployment demonstrating stable occluder position within the interventricular septum (white arrow) with trace residual left-to-right shunting. Abbreviations: LV, left ventricle; RA, right atrium; RV, right ventricle.

Repeat echocardiogram on hospital day 10 revealed persistent LVEF of 40% with apical and inferoseptal wall akinesis despite vasoactive inotropic support. On hospital day 11, the patient underwent percutaneous VSR closure under fluoroscopic and echocardiographic guidance (*Figure 3*). Right heart catheterization (RHC) and left ventriculography demonstrated a Qp:Qs of 2.5, which rapidly improved to 1.6 after deployment of a 24-mm Amplatzer occluder device. Intraprocedural echocardiography confirmed stable device position with minimal residual left-to-right shunting. Repeat intraprocedural RHC showed further reduction in left-to-right shunting with Qp:Qs improving to 1.1 and decreased oxygen-step up from the inferior vena cava to the pulmonary artery compared to pre-closure (*Table 1*). These findings indicated improved haemodynamics and short-term clinical stabilization, permitting IABP removal on hospital day 13. In the days following VSR closure, she developed severe lactic acidosis (>23.0 mmol/L), leukocytosis (22 000 cells/µL), and a mixed venous oxygen content of 60% suggestive of combined septic and cardiogenic shock. A non-contrasted CT of the chest, abdomen, and pelvis did not reveal additional sources of infection outside of pneumonia, and hs-T-troponin remained elevated but stable (1118–1437 ng/L). Repeat echocardiography showed a decreased LVEF of 31% and mild residual shunting near the inferior device rim. Impella CP was placed on hospital day 17 for worsening haemodynamics in the setting of septic shock. Despite added MCS, the patient's condition continued to deteriorate and after discussion with her family, care was transitioned to comfort measures. The patient expired on hospital day 18.

**Table 1:** Pre and post VSR closure haemodynamics and oxygen saturations. Dashed lines indicate missing values

	Date of STEMI		Preprocedure		Postprocedure	
Qp:Qs	−		2.5		1.1	
LVEF %	40		40		45	
	Mean Pressure (mmHg)	Oxygen Saturation %	Mean Pressure (mmHg)	Oxygen Saturation %	Mean Pressure (mmHg)	Oxygen Saturation %
IVC	−	−	12	45	11	58
Right atrium	18	60	12	53	11	53
Right ventricle	25	85	28	−	20	−
Pulmonary artery	40	78	35	62	28	56
Pulmonary wedge	28	−	18	−	15	−
Left ventricle	54	99	45	−	−	−
Aorta	115	99	100	94	−	94

## Discussion

Post-infarct VSR remains one of the most lethal mechanical complications of acute coronary artery occlusion, carrying a mortality rate exceeding 90% when left untreated.^1^ Initial management centres on afterload reduction with pharmacotherapy and MCS to decrease left-to-right shunting, increase cardiac output, and bridge patients to definitive closure.^6^ Surgical repair via patch infarct-exclusion remains standard for larger VSRs (>15 mm), while percutaneous closure is appropriate for smaller VSRs and inoperable patients, offering a shorter hospital length of stay but higher rates of residual shunting. Notably, postoperative outcomes, including 5-year mortality, appear comparable between the two closure methods.^7^

Percutaneous closure was appropriate for our patient given favourable defect anatomy and prohibitive surgical risk. Her VSR was small (4 mm), and its apical muscular location provided adequate surrounding myocardial tissue for secure device anchoring. Furthermore, the patient's clinical condition–including severe pneumonia, hypoxic respiratory failure, increasing sedation requirements, and worsening renal failure–substantially limited her ability to tolerate surgical repair. Management considerations for post-infarct VSR based on defect anatomy and patient clinical status are summarized in *Table 2*. IABP support was initially chosen over Impella CP to minimize device contact with friable septal myocardium and encourage fibrosis before closure. Following VSR closure, Impella CP was placed to assist left ventricular unloading in the setting of worsening septic shock.

**Table 2:** Management considerations for post-infarct VSR based on defect characteristics, haemodynamic status, and surgical risk

Factor	Percutaneous closure favoured	Surgical repair favored	References
Defect size	Small defects < 15 mm, or those with adequate surrounding myocardial rims	Large defects > 15–20 mm, multiple defects, or those with insufficient septal rims for device anchoring	^1–3,7^
Defect location and morphology	Apical (if small) midventricular, or located away from valvular structures	Basal, posterior, or inferior defects, those with complex or serpiginous morphology, and proximity to valvular structures	^1,2,4,7^
Haemodynamic status	Haemodynamically stable patients or those stabilized with pharmacologic therapy and/or mechanical circulatory support	Haemodynamically unstable patients requiring definitive repair without prohibitive operative risk	^4–6^
Operative risk	Patients with prohibitive surgical risk (e.g. advanced age, multiorgan failure, renal dysfunction, respiratory failure, or frailty)	Patients with acceptable operative risk or those requiring concomitant surgical revascularization or structural repair	^3–5^

This case also highlights the clinical dilemma of how long VSR repair can be safely delayed in patients who are hypoxic, intubated, or experiencing multifactorial shock. Competing timelines between myocardial healing and multisystem disease influenced the timing of VSR closure in our patient. Delaying VSR closure was favoured to allow septal infarct fibrosis and myocardial recovery following recurrent ventricular fibrillation arrest. However, prolonged mechanical ventilation ultimately led to pneumonia and progressive septic shock, counteracting this beneficial myocardial healing response.

Despite successful technical deployment of the Amplatzer occluder device and initial improvement in cardiac function allowing for IABP removal, several factors likely contributed to the patient's subsequent cardiac failure and demise. The initial degree of ischaemic injury was extensive, and although cardiac function improved with medical therapy, MCS, and ultimately VSR closure, the already weakened myocardium succumbed to increased metabolic demand and vasoplegia associated with septic shock. Although mild residual peri-device shunting was noted postprocedurally by a Qp:Qs of 1.1 and visually by Doppler flow on echocardiography, this value falls within an acceptable range (Qp:Qs ≤ 1.5) based on reported values.^8,9^ Overall, the patient's irreversible ischaemic injury and septic shock-related myocardial depression were likely the dominant drivers of her decline post-closure.

## Conclusion

This case demonstrates both the potential and limitations of delayed percutaneous VSR closure, emphasizing early recognition, prompt initiation of MCS, and nuanced decisions regarding timing and modality of repair to effectively manage this fatal complication of acute myocardial infarction.

## Lead author biography


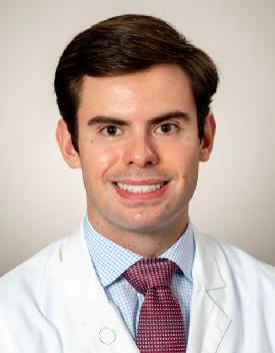


Thomas A. Cook is a third-year medical student at Macon and Joan Brock Virginia Health Sciences Eastern Virginia Medical School at Old Dominion University, with research interests in cardiovascular medicine, structural interventions, and vascular surgery. He has presented multiple case reports and clinical studies at regional meetings and helped establish and lead the Cardiology Interest Group at his home institution as its President. In his free time, he enjoys cooking, travelling, and staying active.

## Supplementary Material

ytag253_Supplementary_Data

## Data Availability

The data underlying this article are included within the article and its Supplementary material. Additional data are not publicly available due to patient confidentiality and privacy considerations.

## Supplementary material

**Consent:** The authors confirm that witnessed verbal consent for submission and publication of this case report including images and associated text has been obtained from the patient’s next of kin. This has been discussed with the editors.
