# Supplementary material for: Percutaneous closure of ventricular septal rupture after myocardial infarction: a case report of competing timelines in multifactorial shock
Source: Eur Heart J Case Rep. 2026 Apr 20;10(4):ytag253. doi: 10.1093/ehjcr/ytag253 (PMC13096803; doi:10.1093/ehjcr/ytag253)
Supplement: ytag253_Supplementary_Data [file ytag253_supplementary_data.pdf]

European
Heart Journal
Case Reports

Lack of written consent form

The Editorial Board of *European Heart Journal – Case Reports* believes in the ethical requirement that patients should consent to the publication of their cases. However, it is also appreciated that there are some circumstances where the ability to gain informed consent is not possible or appropriate. For more details about the importance of patient consent for case reports, see Thomson & Camm, Eur Heart J Case Rep 2021 (doi:10.1093/ehjcr/ytaa560).

This form should be used by authors wishing to submit a case report/case series/grand round/images in cardiology article to *European Heart Journal – Case Reports* where written consent is not available for a patient included in the manuscript.

Please use the lack of written consent flow chart to confirm the action that is required. Please provide the information as detailed as possible.

Manuscript Title: Percutaneous Closure of Ventricular Septal Rupture after Myocardial Infarction: A Case Report of Competing Timelines in Multifactorial Shock

Manuscript ID (if known): EHJ-CR-D-25-01635

Scenario A – (Witnessed) verbal consent to publish has been obtained from the patient but written consent is not possible

A.1. Please outline the reason(s) why written consent from the patient has not been possible in this situation:

Consent for case report was obtained in hospital at completion of successful case by FAMILY. The patient's family was eager to be involved

[illegible]

A.2. Please provide the date of the verbal consent: 5/6/2025 (Procedure Date)

A.3. Please provide the name of the person obtaining verbal consent: Matthew Summers MD (Structural Heart PD)

A.4. Please provide the name and role of the person who witnessed the verbal consent: Deepak Talreja – Clinical chief, cardiology and co-operator

A.5. If verbal consent was not witnessed, please outline the reason(s) why this was not possible:

N/A

Scenario B – The patient is deceased and while there are next-of-kin/surviving relatives, it is not possible to contact them.

B.1. Please outline the reason(s) why the next-of-kin/surviving relative(s) cannot be contacted in this situation (e.g., provide details about which attempts were performed by whom, when, how many times, etc.):

N/A - verbal consent periprocedurally from family

B.2. If the next-of-kin/surviving relatives contact details are not available, please outline what attempts have been made to obtain these (provide as many details as possible: what, by whom, when, how many times,...):

"

Scenario C – The patient is deceased and has no surviving relatives / next-of-kin.

D.1. Please provide details of this scenario and how it has been confirmed that there are no appropriate surviving relatives to contact (please be as precise as possible):

N/A

Scenario D – The patient is alive but has not been contacted to seek consent to publish.

D.1. Please provide details of the steps taken by the authors to obtain contact details for the patient (be as precise as possible, e.g. no contact details for the patient, legal or ethical restrictions, which actions were taken, when, by whom,...):

N/A

I hereby certify that all information provided in this document is complete, true, and accurate to the best of my knowledge. I acknowledge and accept full responsibility for the correctness of these statements, and I confirm that I have undertaken all reasonable and appropriate measures to comply with the applicable ethical standards, including the obligation to seek and obtain informed patient consent.

Date: 12/10/25 Signature: [signature]

Name of the accountable author: Matthew R Summers
